# Value co-creation in healthcare: evidence from innovative therapeutic alternatives for hereditary angioedema

**DOI:** 10.1186/s12913-018-3389-y

**Published:** 2018-07-20

**Authors:** Rosanna Spanò, Nadia Di Paola, Maria Bova, Alessandro Barbarino

**Affiliations:** 1 Department of Economics, Management, Institutions, University of Naples Federico II, Campus Universitario di Monte S. Angelo, Via Cintia 24, 80126 Naples, Italy; 2 Department of Translational Medical Sciences, University of Naples Federico II, Via S. Pansini 5, 80131 Naples, Italy; 3 Department of Public Health, University of Naples Federico II, Via S. Pansini 5, 80131 Naples, Italy

**Keywords:** Value co-creation, Service-dominant logic, C1-INH-HAE, Payer perspective, Societal perspective, Compliance

## Abstract

**Background:**

Our research focuses on the co-creation of value in healthcare with reference to a case of hereditary angioedema with C1 inhibitor deficiency (C1-INH-HAE). Our work is mainly based on the concept of value co-creation in healthcare. The aim of this study is to assess the impact of an alternative treatment strategy – self-administration – by focusing on treatment outcomes and costs to understand if innovative therapeutic solutions can create value for patients and healthcare systems.

**Methods:**

This paper compares home-based and hospital-based therapeutic strategies (independent of treatment type) with a cost minimization analysis. It encompasses compliance issues and focuses on both payer and societal perspectives, also benefiting from an operationalization of the service-dominant logic model for healthcare delivery. Data were collected over a 6-month period (August 2014–January 2015) through monthly patient interviews. Archival data were used for variable measurement.

**Results:**

Thirty-nine out of 62 patients enrolled in the study, experienced at least one HAE attacks, equally distributed between home and hospital-based strategies. No evidence of correlation between therapeutic strategy and disease severity score (*p* = 0.351), compliance (*p* = 0.399), and quality of life (*p* = 0.971), were found. Total direct cost per attack amounts to € 1224 for home-based strategy with respect to € 1454 for hospital-based strategy, with a savings of € 230. The economic advantage of the home-based strategy almost doubles if the societal perspective was considered due to a further savings of €169 (less missed work/school days and no travel expenses).

**Conclusions:**

Our study suggests that home-based therapies represent a feasible strategy for managing C1-INH-HAE and may result in lower costs and increased value for both patients and the healthcare systems. The findings are relevant to the debate on and extend the extant literature to provide a broader view of value co-creation dynamics for home-based therapies in healthcare and their positive effects. The insights are relevant to practitioners and policy makers.

## Background

Economic viability, quality, and accountability are critical to health systems worldwide, considering the increasing survival rates for many diseases. It is well acknowledged that a worldwide challenge to healthcare systems is that of rising costs, scientific, political and economic changes, ethical issues and the demand for greater patient safety and attention to human well-being [1]. Factors such as the globalization, innovation and technologic revolutions that have occurred in recent years have triggered physicians, patients, the public and policy makers to dedicate their attention to issues that relate to quality of life and available treatment options. These pressures have led European countries to introduce many measures to address economic sustainability concerns and satisfy citizens’ calls for greater accountability in the effectiveness and quality of health services [2]. However, the majority of these healthcare systems still struggle to achieve these goals. The current focus is no longer on survival alone but instead on “quality survival” [3], and intense debate continues on possible ways forward.

Such an evolutionary context is characterized by the increasing number of conceived interventions that attempt to fulfill in practice the expectations of patient-centered medicine and that have been realized at different institutional levels. It is currently widely acknowledged that to enhance the quality of healthcare service delivery – and the quality of health systems in general – citizen involvement is paramount. To enhance the quality of the healthcare services delivered, it is necessary to ensure citizen involvement. Citizens are no longer considered mere users of healthcare services but are highly regarded as co-producers of the value that is created [4]. Citizens are also considered co-producers of the increased quality levels that the system can (or should be able to) provide [5, 6]. Citizens, as co-producers of healthcare systems, enable healthcare delivery processes, which are designed according to their needs, to perform better [7, 8].

In this regard, recent contributions [9–12] enhance and enrich the perspective of the co-creation of value (in healthcare, too), inspired by service-dominant logic [13, 14]. Central to these issues is the fact that conflicting logic and conflicting objectives increasingly represent a central worry in healthcare, and co-creation strategies represent a possible way forward toward a system perspective, instead of pursuing single and separate goals to the detriment of others.

On these premises, our research focuses on the co-creation of value in healthcare with reference to the hereditary angioedema with a C1 inhibitor deficiency (C1-INH-HAE). The aim of this study is to assess the impact of an alternative treatment strategy – self-administration – by focusing on treatment outcomes and costs to assess if innovative therapeutic solutions can create value for patients and healthcare systems.

This study compares home-based and hospital-based therapeutic strategies (independently from the type of treatment) with a cost minimization analysis, which refers to data collected during an observational study of adult patients at a referral center in the south of Italy [15]. It includes an evaluation of compliance issues and focuses on both the payer and societal perspectives. It also benefits from an operationalization of the service-dominant logic (SDL) model proposed by McColl-Kennedy et al. [16] for healthcare delivery. Data were collected over a 6-month period (August 2014–January 2015) through monthly patient interviews with the patients. Archival data were used for variable measurement.

Our study suggests that home-based therapies represent a feasible strategy for managing C1-INH-HAE and may result in cost savings and value creation for patients and the entire health system. These findings are relevant to debate and extend the current literature to provide a broader view of value co-creation dynamics for home-based therapies in healthcare and their positive effects. These insights are relevant to practitioners and policy makers.

The remainder of the paper is organized as follows: the second section assesses the prior literature on value co-creation, identifying its healthcare implications, taking into account the increasing importance of SDL, and clarifying these issues with respect to the specific case of C1-INH-HAE. The third section describes the study methods. The fourth reports our findings. Finally, we discuss our results and provide some concluding remarks.

## Literature review

This section aims to provide a framework for this study by briefly reviewing the debate concerning the increasing relevance of value co-creation to healthcare. It highlights several areas of primary importance to the comprehension of the phenomena investigated in general, with special reference to C1-INH-HAE. To this end, this section is divided into two sub-sections.

First, we clarify the conception of value that this paper employs and the implications of a value co-creation logic that focuses on the possible benefits to healthcare. We systematically address multiple and conflicting objectives, also showing an operational model – namely, the service-dominant logic model – to translate such aspirations into practical achievements.

We then present the extant debate on C1-INH-HAE to highlight issues that currently affect both the literature and practice, in turn making an argument that the involvement and empowerment of patients is actually a possible way forward for value co-creation for C1-INH-HAE management.

### Value co-creation and service-dominant logic: Implications for healthcare

As stated above, the fundamental premise of this paper is based on the broad conception of value, which to date is a fundamental imperative for practitioners and researchers in any discipline [17]. *Value* is a chameleon concept that over the last 30 years has constantly evolved, increasingly enlarging its boundaries and content. As Gallarza et al. [17] signal, value is still nebulously defined and subject to future integration. For instance, the very early definition of value that traces back to the 1980s was linked to the trade-off between price and quality. In contrast, more contemporary definitions are rooted in experiential approaches that go far beyond rationalism and encompass symbolism and emotions (for a deeper examination of the implications of perceived value and its emotional dimensions, see [18]). This is even more relevant in a field such as healthcare, where quality of life perceptions gain momentum and are at the forefront of policy-makers’ agendas. With this in mind, the concept of value in our study not only considers the well-acknowledged dimensions of quality of clinical/surgical/therapeutical procedures vs effectiveness and cost containment. Skeptics have often characterized physicians in relation to (unclear) conceptions of value that are featured in the current debate, essentially because they are frequently reduced to cost-reduction pressures [19] or outcome quality demands. We therefore go even farther to take into account dimensions relating to patients’ emotions and feelings during medical therapy.

The actors in the healthcare sector often have conflicting objectives, such as access to services, profitability, high quality, cost containment, safety, convenience, the centrality of the individual and patient satisfaction [4, 20–23]. However, as briefly discussed above, the concepts of *value* and *quality* imply more than simplistic economic or financial savings but the need to reduce expenses while preventing any negative effects on patient care [4, 24]. Clearly, the outcomes in this approach are multidimensional and condition specific. They must be designed according to patient needs and should be monitored over the entire care cycle [4, 25]. These outcomes consider the interactions of many exogenous factors, such as compliance with recommendations, complications, perceived patient satisfaction, and clinical and patient-reported outcomes regarding their perceived well-being [24].

In this domain, the introduction of value co-creation logic is paramount. Such logic is driven by an awareness that *no business is an island*. Value co-creation emphasizes processes that include actions by service providers, customers, and possibly other actors, all-encompassing processes with no distinction between the roles and actions of the above-cited subjects [26, 27]. This is certainly a view that fully applies to the medical field, where cooperative practices between firms (e.g., healthcare organizations and the entire healthcare system) and customers (e.g., physicians, patients and caregivers) can lead to better performance overall, with the customer seen as both a major contributor and a beneficiary [28]. Such cooperative practices – based on dialogue and unique one-to-one interactions (see [29]) – can be the way forward to understanding these aforementioned conflicting objectives while advancing toward the creation of value for all of the stakeholders involved in the process.

What should be noted is that the key to achieving such a multifaceted value is the creation of a common goal that unifies the interests of all involved parties, leading to circumstances in which increased patient value (for example, perceived well-being that improves therapeutic compliance) leads to gains for all subjects involved (for instance, caregivers have a lighter caseload and physicians reduce the risk of adverse events). Consequently, the economic sustainability of the health system is positively affected (for instance, compliance reduces the length of the rescue time, with reductions in direct and indirect expenditures), in turn permitting available additional resources (in the broad sense) to be invested in patient wellbeing (see also [4]). Recent contributions [9–12] enhance and enrich the perspective of value co-creation (in healthcare, too), inspired by service-dominant logic [13, 14]. In this sense, the traditional approach to value creation (goods-dominant logic), which revolves around supply and is based on the dichotomy between supplier and customer, is superseded by a service-oriented approach. The latter refers to a multitude of subjects who actively contribute to the creation of value and bring their resources into a multidimensional co-creation mechanism [30]. From the service-dominant perspective, the customer is not a passive subject, a mere recipient who benefits from the value created by the firm, but rather sits in the middle of the value creation process. The customer is able to actively create value by carrying out his proper activities [31]. Moreover, together with him, other subjects that belong to his service network are able to create value. According to SDL, the value is “in-use” rather than “in-exchange” [26].

Such a view may be beneficial in the healthcare setting by increasing the efficiency of health services, improving health outcomes (e.g., concerning compliance) at any stage of the care cycle, reducing the cost and expenditure of patients, health systems, and society, and increasing patient satisfaction [11, 32–34]. In these cases, the co-creation process integrates the healthcare and external resources connected to the market (e.g., complementary therapies and health providers/companies), to the patient’s private network (e.g., family and friends) and to the patient himself [16, 27]. The patient is able to use his knowledge and skills to help generate value, a process that leads to co-production [35] and can allow him to maximize the benefits to him and improve his quality of life with the most appropriate conduct (e.g., in terms of compliance with treatment [36]).

In particular, a recent piece by McColl-Kennedy et al. [16] offers a crucial operationalization of SDL in healthcare, which is extremely useful for driving any reflections on the field. The authors discuss several case studies that show how the focal firm (i.e., the hospital/the research center), the personnel involved (i.e., physicians and other health professionals), other market-facing sources (i.e., firms/other entities), public sources (i.e., support groups, community groups and the government), private sources (family members, colleagues and friends), and personal sources (customers’ self-generated activities) interact to create value. In this way, these constant and ever-evolving interactions may transform the conflicting objectives between these players into opportunities to create superior value.

Even if the need to involve patients in value co-creation is becoming increasingly clear, the way to realize this requires a better understanding of the concept. In this regard, the debate is far from over because extant studies have only contended that monitoring patient satisfaction [9] and engaging patients in care-pathway design [12] are possible viable solutions [37]. In this regard, crucial issues that can no longer be neglected include ethical dilemmas, the emotional involvement of patients, the need for accessible information, the essential coordinated effort among physicians, specialist centers and patients, the possession of a profound knowledge of the available alternatives, and the call for accepted programs, procedures and indicators to evaluate the quality and costs of services.

In this context, an ever-expanding debate refers to rare diseases and their treatment to find and disseminate solutions for improving the effective use of public resources for healthcare delivery. Bearing this in mind, we take McColl-Kennedy et al.’s concept of operationalization [16] to deepen the issues relating to the co-creation of value [21, 38] in the case of hereditary angioedema with a C1 inhibitor deficiency (C1-INH-HAE). We explore the co-creation of value generated by the active involvement of patients in the treatment of this rare disease through an alternative treatment strategy: self-administration. The aim of this study is to assess the impact of the self-administration of plasma-derived C1-INH (pdC1-INH) by focusing on treatment outcomes and costs. In this way, we hope to understand if innovative therapeutic solutions can create value for patients and the entire healthcare system.

### Value co-creation in C1-INH-HAE

C1-INH-HAE is an autosomal dominant disease caused by a quantitative (type I) or functional (type II) deficiency in the C1-esterase inhibitor, which leads to the dysregulated production of bradykinin [39, 40], a powerful vasodilator and a mediator of capillary leakage. Epidemiologic data show that C1-INH-HAE affects approximately 1 in 50,000 individuals worldwide, with no ethnic or gender preferences [41–44]. The disease is characterized by recurrent attacks of subcutaneous or submucosal swelling in various body sites [39]. Abdominal C1-INH-HAE episodes can be particularly debilitating and are associated with severe pain, while laryngeal episodes can be fatal because of the risk of an airway obstruction [39]. C1-INH-HAE afflicts patients over their lifetime and has a very negative impact on patient quality of life, as emphasized by a cross-sectional study based on a patient-reported outcomes approach, in which the health-related quality of life (HRQoL) from the patient’s (and caregiver’s) perspective was investigated [45]. These findings demonstrate the attack’s wide-ranging impact on the patient’s life due to short-term disability and the long-term effects caused by anxiety and fear between attacks. Furthermore, patients and caregivers experience an interruption in work/school/activity during the attacks [45, 46]. The economic burden of C1-INH-HAE is also considerably higher in affected individuals, which is shown by a survey conducted in the US from 2007 to 2008 that estimated $42,000 in total annual costs for an average hereditary angioedema patient [47]. Moreover, C1-INH-HAE attacks are a frequent cause of ED visits [40, 48].

Due to the lack of awareness of this rare disease and the fact that its manifestations are often indistinguishable from the symptoms of more common angioedema forms, many patients do not receive timely, adequate treatment [49]. A lack of awareness also leads to a delayed diagnosis, as reported by an analysis of a European registry that found that the time between the first swelling episode and disease diagnosis was approximately 8.5 years [50]. This delay commonly led to prolonged episodes, increased severity, and hospitalization [51]. On this basis, since the publication in 2003 of the first consensus document on hereditary angioedema therapy [52], efforts have been made to improve the recognition and management of C1-INH-HAE. A crucial role is played by referral centers, which provide access to expert medical advice, patient education, and specialist treatment with patient-centered integrated care. Moreover, in the last 10 years, the availability of an effective treatment for acute attacks and appropriate prophylaxis has contributed to the improvement of the individual, societal and economic consequences of the disease [46]. A recent study [53] compared the perceived quality of life data of 134 patients and found that quality of life significantly improved in almost all domains from 2009 to 2015. However, a significant burden of illness remains.

In this regard, the concept of self-administration is relevant, as it allows for the timely treatment of acute attacks [46, 51, 54–56]. Timely intervention reduces the duration of the attacks, reduces recovery times and increases the quality of life of patients and caregivers [46, 57–59]. Bygum [60] found that most patients who learn how to perform home therapy reported a significant improvement in the psychological and physical impact of the disease. Home therapy is also associated with a decrease in the number of hospitalizations and ED visits and a reduction in missed work/school days for patients and caregivers [32, 57, 60–63]. Consequently, home therapy results in cost savings for both the payer and society at large [32, 62]. Despite its value and the recommendations set forth by guidelines and consensus documents [51, 54, 64–66], several barriers to self-administration still exist, which are indicated by a recent survey study designed to assess the current practice of self-administration across Europe, Canada and the United States [55]. These barriers derive from difficulties in administration, a shortage of nursing resources, the patients’ mental capacity, the retaining of skills in the setting of a low attack frequency, and the reluctance among physicians to prescribe self-administration. The slow uptake of self-administration has also been emphasized by guidelines and hereditary angioedema expert meetings that identified training (both for healthcare staff and patients/caregivers) and follow-up as the key factors necessary for encouraging the uptake of this treatment option [51, 56, 58].

**Fig. 1:**
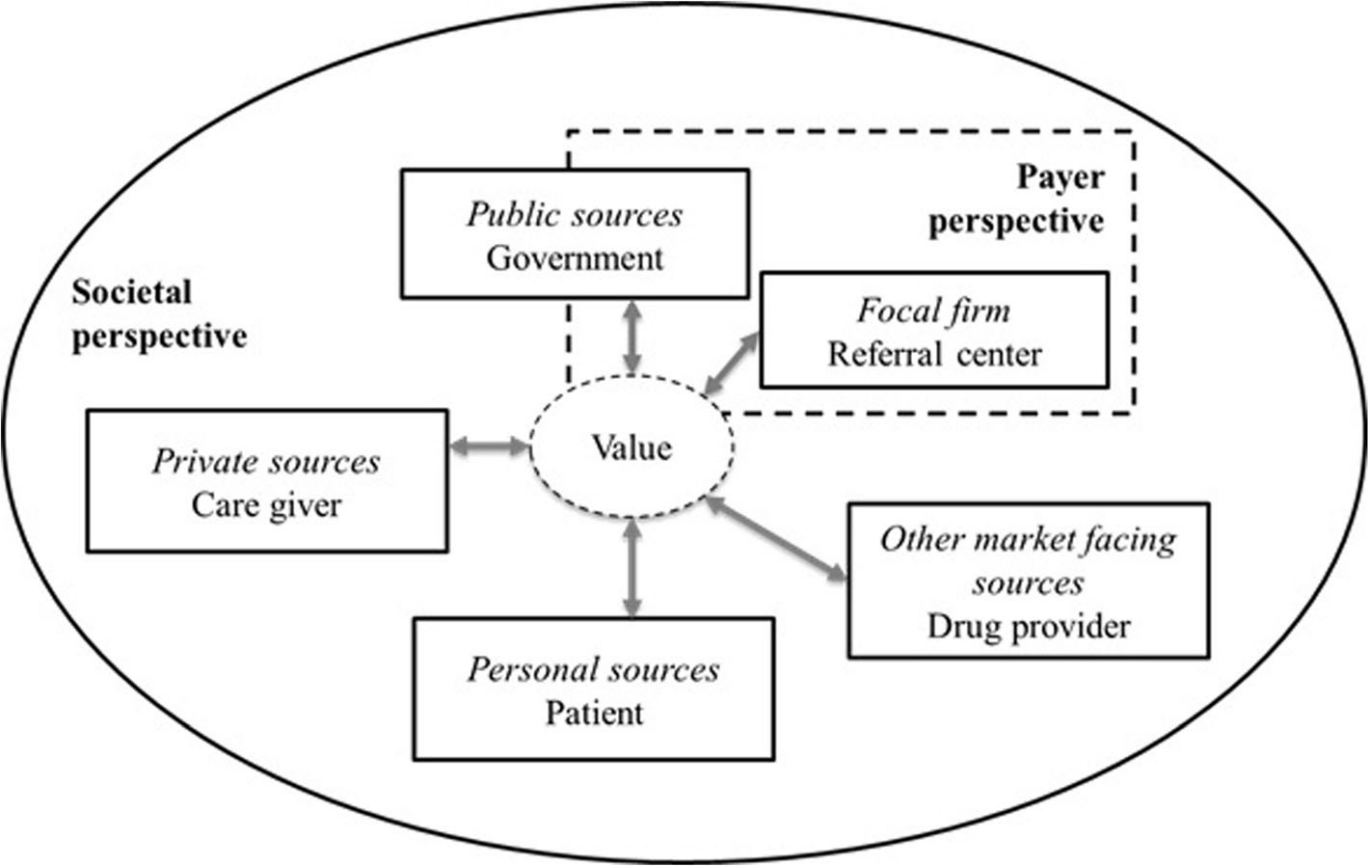
An operationalization of value co-creation in C1-INH-HAE

This description reveals that the extant findings, although incredibly important, still remain at a general level and focus only on the shift from a hospital- to a home-based treatment. Systematic comprehension of issues that involve the impact of home-based strategies compared with hospital-based strategies, such as compliance with a specific therapeutic protocol, is still under-investigated. In contrast, to comprehend whether the involvement and empowerment of patients is actually a possible way forward for value co-creation (that is, the savings per attack from both the payer and societal perspectives, see [32, 62] in the management of C1-INH-HAE, we also consider compliance questions to ensure a more holistic approach to the phenomenon, a concept that has been neglected to date. In addition, to fully capture the dynamics of value creation and the impact it has on the subjects involved in terms of the satisfaction of their conflicting objectives, we perform an analysis that matches the payer and societal perspectives with the SDL model put forth by McColl-Kennedy et al. [16]. We borrow McColl-Kennedy et al.’s categorization of the actors involved (namely, the focal firm, other market-facing sources, public sources, private sources and personal sources) in value co-creation in healthcare [16] and operationalize it for C1-INH-HAE, as reported in Fig. 1. According to this model, the payer perspective allows us to understand the value created for the focal firm (i.e., the referral center) and part of the value created for public sources (the National Health Service). On the other hand, the societal perspective explains the remaining value created for the public sources as well as the value created for other market-facing sources (the drug provider), private sources (the caregiver), and personal sources (the patient).

## Methods

This study represents a continuation and a further step of an observational study [15], approved by Ethics Committee of Università “Federico II” (Naples, Italy), that was conducted over a 6-month period (August 2014–January 2015) and included type I and type II C1-INH-HAE patients treated in a single center in Italy (Naples). Two treatments were available: icatibant (Firazyr®) administered at home by subcutaneous injection (group 1), and pdC1-INH concentrate administered by intravenous infusion both at home (Berinert® or Cinryze®, group 2) or at hospital (only Berinert®, group 3). For the purposes of this paper we drew from the data collected through the above-described observational study [15].

Regarding icatibant home-therapy, physicians teach their patients self-administration at the time of their first prescription. pdC1-INH concentrates are administered by intravenous infusion. The study center started a training program funded by the drug providers (see [32]) in 2010. During the training, thanks to a simulator arm, patients also were taught how to cope with cases of laryngeal attack and the related impossibility of administering the treatment. Over the period August 2014–January 2015 62 patients were in treatment at the referral center of the current study. All of them were visited at baseline and followed up for 6 months, recurring to phone interviews at least one time per month. At the beginning, during the first dialogue, the patients were told to keep a timely and full record of the characteristics of each attack and the related treatment, as well as to take note of any relevant features. The monthly interviews allowed us to timely collect data, especially concerning demographic characteristics, the number of attacks in the previous month, the number of treated attacks, the number of vials used for each attack, the number of ED visits, and the number of missed workdays. Disease severity was also explored relying upon the general disease severity score^1^ developed by Bygum et al. [67].

Results of Squeglia et al. [15] have shown no statistical difference in quality of life between treatment groups (measured using HAE-QoL questionnaire); furthermore, the disease severity score was significantly correlated with quality of life (*p* = 0.008), but no correlation resulted between disease severity and treatment strategy (*p* = 0.74) or compliance (*p* = 0.32). Such evidences suggest to compare home-based therapy (strategy 1) with hospital-based therapy (strategy 2) by employing a cost-minimization analysis, since these different medical interventions seems to provide the same therapeutic effects. Accordingly, patients who did not report at least one treated attack during the observation period (17) were excluded from the cost-minimization analysis.

We therefore look at cost-related differences and their impact in terms of created value. For the abovementioned strategies, we assessed the impact of self-administration on the costs that relate to the management of acute C1-INH-HAE attacks from both the Italian healthcare payer perspective and the societal perspective.

For each of the above-mentioned groups, namely, 1) home-based therapy with icatibant, 2) home-based therapy with pdC1-INH concentrate, and 3) hospital-based therapy with pdC1-INH concentrate (Berinert®), the first step of the Cost-minimization analysis was to identify the number of attacks treated with each therapeutic strategy (strategy 1 – home-based compared with strategy 2 – hospital-based). For example, for the icatibant and pdC1-INH home-based groups, the attacks treated at home were compiled to quantify compliance costs, while the attacks treated at the hospital or not treated at all were compiled to quantify non-compliance costs. This categorization was useful to identify which types of costs should be included in our calculations and to consider payer and societal perspectives. From the payer perspective, we consider only medical costs, including drug costs and costs that involve ED visits/hospitalization. The societal perspective encompasses medical costs plus non-medical costs, which include missed work/school days (for patients and caregivers) and travel expenses for the ED visits. See Table 1 for a summary of the computation method used.

**Table 1:** Categories for computation

	Payer perspective		Societal perspective		
	Drug costs	ED access/hospitalization	Missed work days (patient)	Missed work days (caregiver)	Transport costs
Strategy 1 (home-based)					
Compliance costs	Always	Never	Quantified according to the time of resolution declared in the case of compliance	Only when declared by patients and according to the time of resolution declared in the case of compliance	Never
Non-compliance costs	Only in the case of attacks treated at the hospital	Only in the case of attacks treated at the hospital	Quantified according to the time of resolution declared in the case of non-compliance	Only when declared by patients and according to the time of resolution declared in the case of non-compliance	Only in the case of attacks treated at the hospital
Strategy 2 (hospital-based)					
Compliance costs	Always	Always	Quantified according to the time of resolution declared in the case of compliance	Only when declared by patients and according to the time of resolution declared in the case of compliance	Always
Non-compliance costs	Never	Never	Quantified according to the time of resolution declared in the case of non-compliance	Only when declared by patients and according to the time of resolution declared in the case of non-compliance	Never

The above-cited costs were measured as follows. Drug costs were calculated as the number of vials multiplied by the unitary cost of the vial. The cost of ED admission was estimated to equal €18.59 and included only the first evaluation. Any additional clinical examinations were considered only when declared and according to the fees of the Regione Campania for 2013 and the Diagnosis Related Groups (DRGs) of the Italian National Healthcare System (see [32]). Travel expenses were calculated on the basis of the distance reported by patients/caregivers and the tariffs per km reported by Automobile Club Italia [68]. The cost of missed workdays was measured according to the occupation of the patients/caregivers and was quantified according to the net hourly wage [69]. See Table 2 for a summary of this measurement.

**Table 2:** Measurements

Drug costs			
	Commercial name	Unitary cost (€)	Source
Icatibant	Firazyr®	1490	[72]
Plasma-derived C1 inhibitors	Berinert®	557	[73]
	Cinryze®	1200	[74]
ED admission/hospitalization			
	€		Source
First visit	18.59		[32]
Blood sample	43.09		
Chest X-ray	15.49		
ECG	9.97		
Echo-cardio	41.32		
Echo-abdomen	60.43		
Missed work days			
	€/hour		Source
Net hourly wage for the manufacturing sector	9.62		[69]
Net hourly wage for the construction industry	8.97		
Net hourly wage for the third sector	12.80		
Net hourly wage for the service sector	10.27		
Transport costs			
	€/km		Source
Tariff per km	0.49		[68]

Finally, Quality-Adjusted Life-Years (QALYs) were estimated by applying utility weights reported in the HAE-BOIS-Europe survey [70] for two health states: 0.444 during an attack (the period of time before the onset of symptom relief), and 0.722 following recovery from the attack (after onset of symptom relief), independently of the type of therapy^2^, and comparable across countries. Moreover, utility during an HAE attack is detailed according to attack severity (0.613 for no pain or mild, 0.467 for moderate and 0.080 for severe attacks).

More specifically, time until resolution of treated attacks was evaluated using the utility value 0.444, while time between two consecutive attacks was evaluated using the utility value 0.722; 3 scenarios were investigated to evaluate time until resolution of no-treated attacks since no information about severity were available. Scenario A: all no-treated attacks were assumed no affecting quality of life, i.e. 0.722 utility value was used; Scenario B: all no-treated attacks were assumed no pain or mild, i.e. specific 0.613 utility value was used; Scenario C: no-treated attacks were assumed equal to treated attacks i.e. same 0.444 utility value was used.

Consistently with previous considerations, we do not expect differences in QALY between the two strategies since difference in quality of life did not resulted significant in the first analysis by Squeglia et al. [15]. However, this calculation might be useful since QALYs allow capturing the net impact of the effects of a condition.

### Statistical analysis

As declared above, this analysis involves a subgroup of patients investigated in the previous observational study, hence the same statistical analyses were performed in order to assess the equivalence of efficacy between home-based and hospital strategies. The Pearson χ^2^ test was used to evaluate correlation between treatment strategy and the same clinical factors included in Squeglia et al. [15]: age, age at diagnosis, sex, level of education, disease severity score, number of attacks, and the quality of life. Furthermore, differences in quality of life, expressed in QALYs, between home-based and hospital strategies, were compared using the non-parametric Wilcoxon test. Statistical analysis was performed using IBM SPSS Statistics software, statistical significance is defined as a *p*-value lower than 0.05.

## Results

Out of the initial sample of 62 patients, six were lost to follow-up and 17 report no treated attacks during the observation period, which resulted in a final population of 39 patients (66.6% female, mean age 33.7 years). According to the treatment that they received, the patients were divided into the following three groups: 20 patients were treated with home-based pdC1-INH concentrate (65.0% female, mean age 33.0 years); 11 patients were treated with home-based icatibant (72.7% female, mean age 37.0 years); and 8 patients were treated with hospital-based pdC1-INH concentrate (62.5% female, mean age 31.0 years). The mean severity score was 7.8, 6.9 and 6.6 for patients who received home-based pdC1-INH, home-based icatibant and hospital-based pdC1-INH, respectively. The characteristics of the study population and treatment groups are summarized in Table 3.

**Table 3:** Study population and treatment group characteristics

	Home-based pdC1-INH (*N* = 20)	Home-based icatibant (*N* = 11)	Hospital-based pdC1-INH (*N* = 8)	Overall (*N* = 39)
Gender, N (%)				
Female	13 (65.0)	8 (72.7)	5 (62.5)	26 (66.6)
Male	7 (35.0)	3 (27.3)	3 (37.5)	13 (33.4)
Age, mean (±SD)	33.0 (±18.7)	37.0 (±11.7)	31.0 (±20.4)	33.7 (±17.1)
Disease severity score, mean (±SD)	7.8 (±1.7)	6.9 (±1.6)	6.6 (±1.5)	7.3 (±1.7)

*pdC1-INH* plasma-derived C1-esterase inhibitor concentrate, *SD* standard deviation

Our analysis revealed that in the icatibant group, 188 attacks occurred over the study period, of which only 84 were treated in compliance with a home-based strategy, requiring the consumption of 93 vials. The rest of the attacks (104) were not treated at all because no ED access was reported. In the pdC1-INH home-based group, a total number of 556 attacks occurred over the study period, of which 413 were treated in compliance with the strategy, 22 were treated in the ED and the rest were not treated at all. The consumption of vials for this group was 765 during compliant treatment and 45 during hospital treatment. In the pdC1-INH hospital-based group, 54 attacks occurred over the study period, 26 of which were treated, requiring the consumption of 67 vials. The characteristics of the attacks in these three groups of patients are summarized in Table 4.

**Table 4:** The number of attacks and the treatment characteristics in the three groups of patients

	Number of attacks	Number of treated attacks (compliant and non-compliant)	% of treated attacks	Number of vials	Average number of vials per attack
Strategy 1 (home-based)					
Icatibant	188	84	44.68%	93	1.11
PdC1-INH	556	435	78.24%	810	1.86
Strategy 2 (hospital-based)					
Hospital-based PdC1-INH	54	26	48.15%	67	2.58
Total Strategy 1	744	519	69.76%	903	1.74
Total Strategy 2	54	26	48.15%	67	2.58

*pdC1-INH* plasma-derived C1-esterase inhibitor concentrate

As expected, no statistically significant differences can be seen between patients receiving the two strategies, with regard to age (*p* = 0.182), age at diagnosis (*p* = 0.476), level of education (*p* = 0.867), and sex (*p* = 0.685). Instead, there is a correlation between the therapeutic strategy and the number of attacks (*p* = 0.034), indicating that increasing number of attacks led to opt for home-based therapy.

Consistently with the previous study [15], even in this subpopulation there is no evidence of correlation between therapeutic strategy and disease severity score (≥ 7 [severe disease] or < 7 [mild to moderate disease], *p* = 0.351), compliance (*p* = 0.399), and quality of life (*p* = 0.971).

Table 5 reports the costs of the compliant treatment for the two considered strategies and provides details regarding medical and non-medical costs that focus on the average values per patient and per attack. A first interesting issue pertains to transport costs. These costs are completely absent in strategy 1 and not relevant in strategy 2. Likewise, ED access costs do not heavily impact the strategy choice.

**Table 5:** Costs of attacks treated in compliance with the strategies

	Strategy 1 (home-based)			Strategy 2 (hospital-based)			Savings per attack
	Total	Per patient (*N* = 31)	Per attack (*N* = 497)	Total	Per patient (*N* = 8)	Per attack (*N* = 26)	
Medical costs (€)							
Drug cost	608,399.00	19,625.77	1224.14	37,319.00	4664.88	1435.35	
ED visits	0	0	0	483.34	60.42	18.59	
Total	608,399.00	19,625.77	1224.14	37,802.34	4725.29	1453.94	
Non-medical costs (€)							
Missed work/school days	13,737.47	443.14	27.64	5071.65	633.96	195.06	
Travel expenses	0	0	0	39.74	4.97	1.53	
Total	13,737.47	443.14	27.64	5111.39	638.92	196.59	
Payer perspective (€)	608,399.00	19,625.77	1224.14	37,802.34	4725.29	1453.94	229.8
Societal perspective (€)	622,136.47	20,068.92	1251.78	42,913.73	5364.22	1650.53	398.74

Compliance with strategy 1 eliminates all medical costs except for those related to drug consumption. However, the savings per attack (i.e., savings from the payer perspective) were only 15.83% per attack when comparing the two st.

rategies. Compliance with strategy 1 lowered the total non-medical cost, with a savings of 85.9% per attack compared with hospital-based treatment. In particular, transport costs were completely eliminated, and missed workday costs were lowered by 85.83%. Consequently, the savings per attack were higher from the societal perspective than from the payer perspective and amounted to 24.15%.

In addition, it is worth considering that when providing details about the payer and the societal perspectives, these findings allow us to capture the value created for the various players involved in these two strategies within the SDL model. Table 6 presents the value co-creation dynamics that occur for each strategy, describing the objectives, the activities carried out, the type of participation in the value-creation process, and the value obtained.

**Table 6:** Value co-creation in C1-INH-HAE

		Strategy 1 (home-based)			Strategy 2 (hospital-based)		
Actors	Objectives	Activities	Partecipation to the value creation process	Value obtained	Activities	Partecipation to the value creation process	Value obtained
Focal	Cost vs quality	Offers the therapy at hospital	Active	(increased)public health due to increased compliance, but less medical costs per attack other than drugs due to treatment at home	Offers the therapy at hospital	Active	Public health
Other market facing	Sales	Provides the drug and the training for patients and caregivers	Active	(increased) profits due to increased compliance	Provides the drug	Active	Profits
Public	Cost vs quality	Funds the focal firm	Active	(increased) public health due to increased compliance, but less medical costs per attack due to treatment at home. (increased) social value due to less non-medical costs per attack	Funds the focal firm	Active	Public health
Personal	Quality	Involved in self-administration	Active	Value exceeding quality of care and allowing better life and social conditions	Receives the therapy	Recipient	Care
Private	Quality	Involved in self-administration	Active	Value exceeding quality of care of the assisted patient and allowing better life and social conditions	Assists the patient	Recipient	Care of the assisted patient

Table 6 assumes that the objectives of each subject are common to both strategies but can be pursued through different routes. Starting from the patient’s initial involvement, in strategy 1, all players are actively involved in the value creation process, whereas in strategy 2, only 3 players are actively involved, and patients and caregivers are simply recipients of the value created. The result of such a situation is a different ability to satisfy the objectives of all actors. Strategy 2 permits only a partial satisfaction of the objectives of the subjects involved (especially in the case of public sources and the focal firm, for which the realization of quality outcomes in terms of increased public health due to compliance has no effect on cost containment) and without any systematic interaction. This means that we recognize different types of value created separately and are not able to create a superior value at a system level. In contrast, strategy 1 allows us to detect the broadest satisfaction of the objectives and a positive interaction at the system level that produces a superior value for all players and for the system as a whole.

**Fig. 2:**
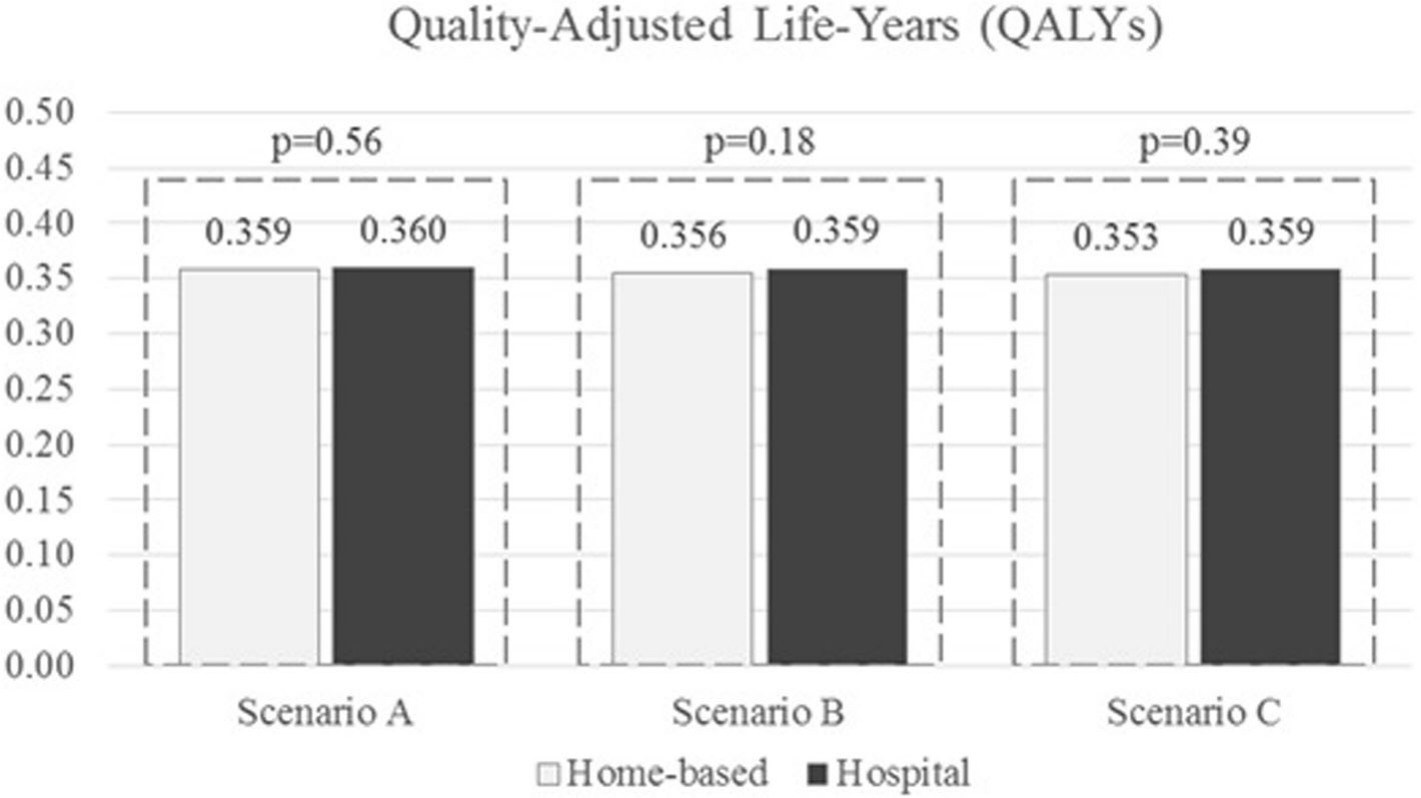
Comparison between home-based and hospital quality of life evaluated according to different assumption on no-treated attack utility. *Note.* Scenario A: no-treated attack utility equals to no-attack utility; Scenario B: no-treated attack utility equals to no pain or mild attack; Scenario C: no-treated attack utility equals to treated attack utility

Quality of life for both home-based and hospital strategies results around 0.35–0.36 QALY (Fig. 2). There is no evidence of statistically significant differences between the two strategies in all the three scenarios (*p* > 0.05); furthermore QALYs are absolutely comparable in both alternatives with an absolute difference of at most 0.005 in Scenario C. We observe that, since utility weights range between 0 (death) and 1 (perfect health), the maximum value of quality of life during the study period could be 0.5 QALY; hence we can conclude that patients living with HAE suffer a reduction in quality of life of almost 30%.

## Discussion

This study was commenced after acknowledging that crucial concerns in healthcare come from conflicting logics and conflicting objectives and that value co-creation approaches represent a possible way forward toward a system perspective, instead of pursuing single and separate goals to the detriment of others. On this basis, recognizing the increasing importance of rare diseases and their impact, this study aimed to deepen the issues relating to the co-creation of value [21, 38] in patients with hereditary angioedema and C1 inhibitor deficiency (C1-INH-HAE). We explored the co-creation of value generated by the active involvement of patients in the treatment of this rare disease through an alternative treatment strategy: self-administration. The aim of our analysis was to assess the impact of the self-administration of plasma-derived C1-INH (pdC1-INH) in terms of the value created for all parties involved. We showed that a home-based strategy allows us to involve all the players in the value co-creation process, thereby producing a superior value at the system level. Our findings provide an interesting insight from many perspectives.

First, our article adds to the extant literature on the positive impact of self-administration and proposes a broader interpretation of the phenomenon that is not strictly related to the specific treatment type. This article also considers issues related to compliance, which have been neglected to date. Indeed, research to date has not encompassed compliance issues and focuses only on the specific type of treatment, thereby offering only a partial view of the value co-creation effect. More specifically, a study conducted in Spain that compared the self-administration of icatibant with hospital administration [62] showed an average savings of €89.8/attack from the payer perspective and €121.30/attack from the societal perspective. In Italy, a recent observational study assessed the treatment outcomes and costs associated with the shift to home therapy with pdC1-INH [32]. Their results noted a significant reduction in hospitalizations and missed work/school days compared with hospital drug administration and mean annual cost decreases from €30,010.57 to 26,621.16/patient and from €29,309.34 to 26,522.04/patient from the societal and payer perspectives, respectively. Our analysis demonstrates that compliance with home-based therapy eliminates all medical costs except the costs of drug consumption. Comparing the two strategies, the savings per attack from the payer perspective amounted to €229.8/attack, and the savings from the societal perspective amounted to €398.74/attack. Compliance with an innovative home-based strategy is economical and affordable from any point of view, but its social importance goes beyond the numbers. According to our results concerning the reduction of missed work days (missed work day costs were lowered by 85.83% with the home-based therapy), the impact of home-based therapy compliance not only depended on cost reduction (+ 24.15% of savings per attack from the societal perspective) but also involved the outcome achieved [4]. Self-administration seems to be a good example of value co-creation in which all of the involved actors can benefit while the economic sustainability of the healthcare system is considered (see also [71], who mention the positive economic impact of self-administration in the treatment of hemophilia in Portugal).

Second, another interesting implication relates to the chance that patients’ active involvement favors their compliance with therapies. These findings even reinforce the issues highlighted by Timmerman et al. [37] advancing the importance of the involvement of users in medical processes by means of innovative tools, thus supporting the co-creation of value in healthcare. Our findings confirm that more research on these issues should be performed, fixing an important starting point for new reflections in the field.

Third, our findings even reinforce the debate on SDL and value co-creation (e.g., [9–14, 26, 30, 31]), adding empirical substance to this theoretical model and complementing it (especially for healthcare adaptations, [16]) with interesting insights rooted in the Italian healthcare field, but expandable and adaptable to different settings. Indeed, our findings allow us to identify the way in which a superior value is created in a complex setting dominated by conflicting logics, showing a possible pathway toward avoiding a hierarchy of objectives and moral hazard, which may result in broad systemic advantages. In this sense, self-administration unveils the synergistic effects of the service-dominant approach in healthcare, which enhances the role of interactions between different value sources (the focal firm, market-related sources, public and private sources) and the potential of self-produced activities (personal sources) in order to generate superior value at the system level.

All the above contributions are not limited to the theoretical domain but have undeniable importance in practice, especially for policy-makers and the multiple subjects operating in health-related fields.

## Conclusions

This paper considers the economic and social impact of two alternative therapeutic strategies. By employing a cost minimization analysis, our study suggests a possible way to create superior and shared value through co-creation approaches. We conclude that home-based therapies represent a feasible strategy for managing C1-INH-HAE and may result in cost savings and value creation for patients as well as the entire health system. A potential limitation of this study is its small sample size. However, C1-INH-HAE is a rare disease, and the enrollment of large patient populations is extremely difficult. Moreover, the abovementioned savings may vary if non-compliant treatments are introduced into our analysis. However, given that the aim of this study was to enhance the potential of patient engagement in therapies to produce a shared value, this option is not considered a relevant one. On this basis, our conclusion is that although several limitations still affect the employment of self-administration in the management of acute C1-INH-HAE attacks, this study describes a positive experience of value co-creation in healthcare that can enhance healthcare delivery. The value created for patients increased, and each subject benefitted both socially and economically. Our findings have interesting and novel implications on both the economic and social impacts of a newer therapeutic option. Our results also confirm the importance of patient involvement in the healthcare delivery process to improve the performance of the healthcare system as a whole.

## Abbreviations

C1-INH-HAE: Hereditary angioedema with C1 inhibitor deficiency
DRGs: Diagnosis Related Groups
ED: Emergency department
HRQoL: Health-related quality of life
pdC1-INH: Plasma-derived C1-inhibitor
SD: Standard deviation

## Footnotes

1 This score ranges from 0 to 10 (from the lowest to the highest disease severity) and is based on the patient’s age at disease onset, the number of organs ever affected, and the need for long-term prophylaxis: age at onset 0–5 years (3 points), age at onset 6–10 years (2 points), age at onset, 11–20 years (1 point), age at onset > >20 years (0 points); skin edema ever (1 point); painful abdominal edema ever (2 points); laryngeal edema ever (2 points); other clinical manifestations (1 point); long-term prophylaxis (1 point).

2 C1-INH concentrate occupies the same clinical position as icatibant; there is no head-to-head clinical evidence to suggest that one of these interventions is more effective than the other, so neither is generally indicated as a first line option (although perceptions of superiority of one or the other may exist among clinicians). Thus, for the treatment comparison, the clinical claim is that C1-INH concentrate is non-inferior to icatibant in efficacy and safety for acute HAE attacks in adults and adolescents. This is based on the ASCIA management algorithm, which recommends either icatibant, or C1-INH concentrate for acute attacks. The appropriate economic evaluation would be a cost-minimization analysis. See both Australian and German Guidelines.
